# Crystal structure of 5-(5,6-di­hydro­benzo[4,5]imidazo[1,2-*c*]quinazolin-6-yl)-2-meth­oxy­phenol

**DOI:** 10.1107/S2056989015021180

**Published:** 2015-11-21

**Authors:** Farook Adam, Md. Azharul Arafath, A. Haque Rosenani, Mohd. R. Razali

**Affiliations:** a School of Chemical Sciences, 11800, USM Pulau Pinang, Malaysia

**Keywords:** crystal structure, cyclization, imidazole derivative

## Abstract

In the mol­ecule of the title compound, C_21_H_17_N_3_O_2_, the 5,6-di­hydro­benzimidazo[1,2-*c*]quinazoline moiety is disordered over two orientations about a pseudo-mirror plane, with a refined occupancy ratio of 0.863 (2):0.137 (2). The dihedral angles formed by the benzimidazole ring system and the benzene ring of the quinazoline group are 14.28 (5) and 4.7 (3)° for the major and minor disorder components, respectively. An intra­molecular O—H⋯O hydrogen bond is present. In the crystal, mol­ecules are linked by O—H⋯N hydrogen bonds, forming chains running parallel to [10-1].

## Related literature   

For the structure of related *N*-heterocyclic Schiff base compounds, see: Cheng *et al.* (2006 ▸); Ünver *et al.* (2010 ▸); Gurumoorthy *et al.* (2010 ▸); Natarajan & Mathews (2011 ▸); Alliouche *et al.* (2014 ▸).
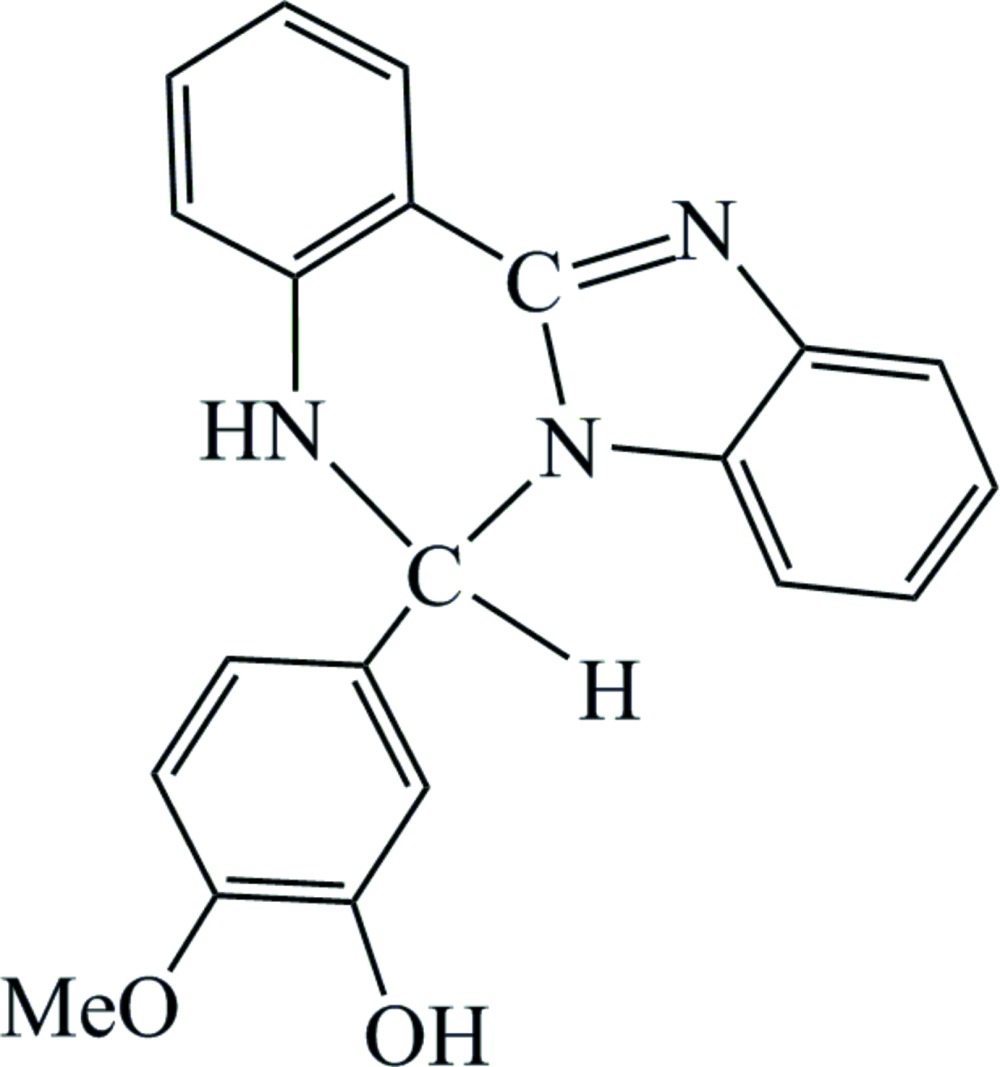


## Experimental   

### Crystal data  

C_21_H_17_N_3_O_2_
*M* *_r_* = 343.38
Monoclinic, 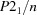

*a* = 9.7359 (7) Å
*b* = 10.0822 (7) Å
*c* = 17.4624 (13) Å
β = 94.2958 (15)°
*V* = 1709.3 (2) Å^3^
*Z* = 4
Mo *K*α radiation
μ = 0.09 mm^−1^
*T* = 294 K
0.29 × 0.20 × 0.12 mm

### Data collection  

Bruker APEXII CCD diffractometer
19177 measured reflections
4987 independent reflections
3575 reflections with *I* > 2σ(*I*)
*R*_int_ = 0.030

### Refinement  

*R*[*F* ^2^ > 2σ(*F* ^2^)] = 0.049
*wR*(*F* ^2^) = 0.139
*S* = 1.05
4987 reflections
370 parameters
752 restraints
H atoms treated by a mixture of independent and constrained refinement
Δρ_max_ = 0.34 e Å^−3^
Δρ_min_ = −0.23 e Å^−3^

### 

Data collection: *APEX2* (Bruker, 2008 ▸); cell refinement: *SAINT* (Bruker, 2008 ▸); data reduction: *SAINT*; program(s) used to solve structure: *SHELXS97* (Sheldrick 2008 ▸); program(s) used to refine structure: *SHELXL2013* (Sheldrick, 2015 ▸); molecular graphics: *SHELXTL* (Sheldrick, 2008 ▸); software used to prepare material for publication: *SHELXTL*.

## Supplementary Material

Crystal structure: contains datablock(s) I, New_Global_Publ_Block. DOI: 10.1107/S2056989015021180/rz5173sup1.cif

Structure factors: contains datablock(s) I. DOI: 10.1107/S2056989015021180/rz5173Isup2.hkl

**Fig. 1.**
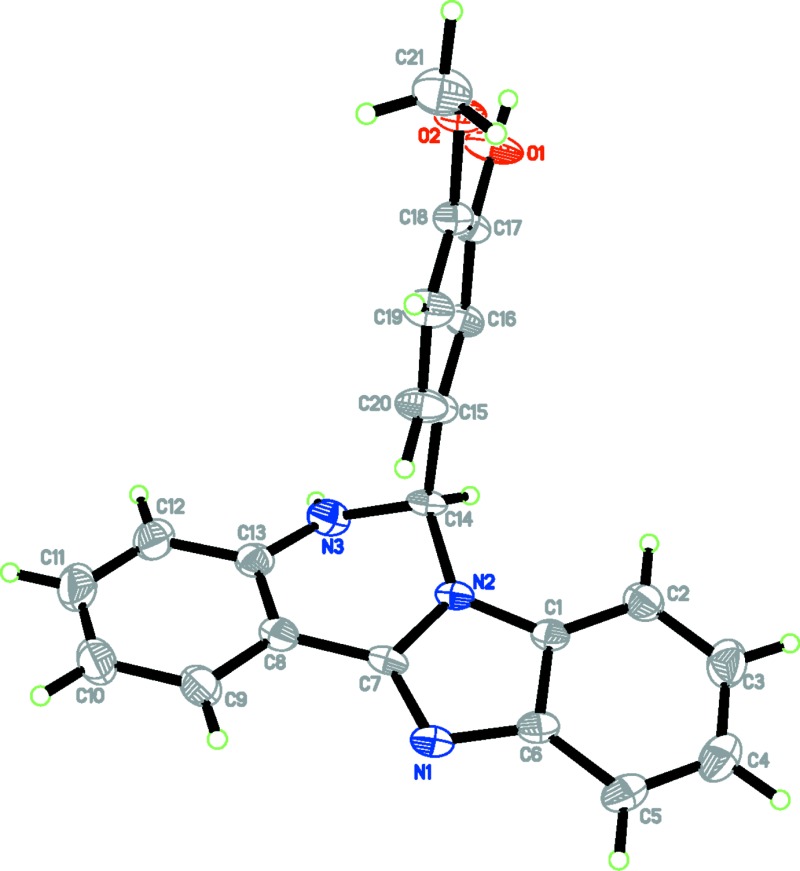
The molecular structure of the title compound with displacement ellipsoids drawn at the 30% probability level. Only the major component of the disordered 5,6-dihydrobenzimidazo[1,2-c]quinazoline moiety is shown.

**Fig. 2.**
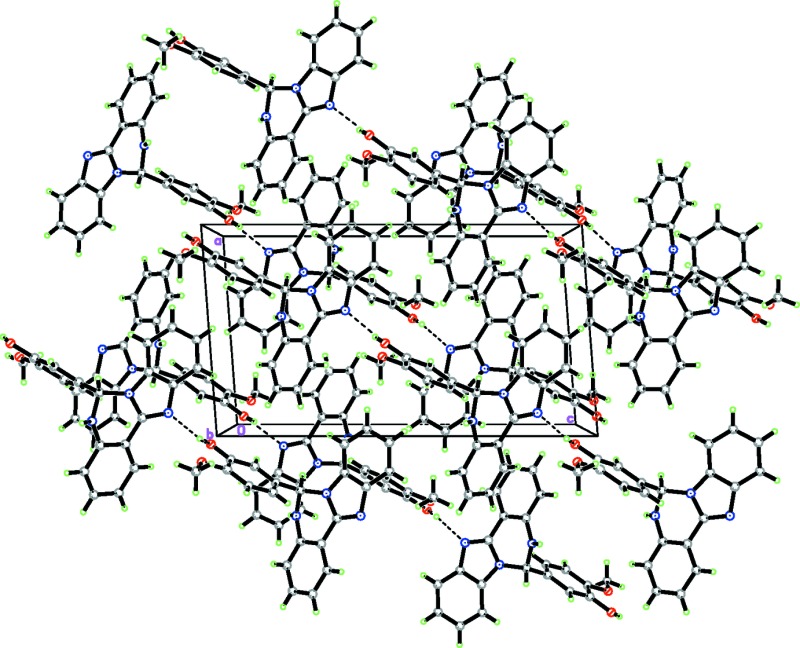
Crystal packing of the title compound viewed down the b axis. Intermolecular hydrogen bonds are shown as dashed lines. Only the major component of the disordered 5,6-dihydrobenzimidazo[1,2-c]quinazoline moiety is shown.

Supporting information file. DOI: 10.1107/S2056989015021180/rz5173Isup3.cml

c . DOI: 10.1107/S2056989015021180/rz5173fig1.tif
The mol­ecular structure of the title compound with displacement ellipsoids drawn at the 30% probability level. Only the major component of the disordered 5,6-di­hydro­benzimidazo[1,2-*c*]quinazoline moiety is shown.

b c . DOI: 10.1107/S2056989015021180/rz5173fig2.tif
Crystal packing of the title compound viewed down the *b* axis. Inter­molecular hydrogen bonds are shown as dashed lines. Only the major component of the disordered 5,6-di­hydro­benzimidazo[1,2-*c*]quinazoline moiety is shown.

CCDC reference: 1048554

Additional supporting information:  crystallographic information; 3D view; checkCIF report

## Figures and Tables

**Table 1:** Hydrogen-bond geometry (Å, °)

*D*—H⋯*A*	*D*—H	H⋯*A*	*D*⋯*A*	*D*—H⋯*A*
O1—H1*O*1⋯O2	0.80 (3)	2.25 (3)	2.6706 (16)	112 (3)
O1—H1*O*1⋯N1^i^	0.80 (3)	1.98 (3)	2.703 (2)	150 (3)
O1—H1*O*1⋯N1*X* ^i^	0.80 (3)	2.18 (3)	2.873 (9)	145 (3)

Symmetry code: (i) 
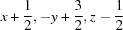
.

## supplementary crystallographic information

### S1. Comment

The title compound (Fig. 1), which features a cyclic structure instead of the planned imine C═N double bond of a Schiff base compound, was unexpectedly obtained by the reaction of 3-hy­droxy-4-meth­oxy-benzaldehyde and 2-(2-amino­phenyl)-1H-benzimidazole. In the molecule, the 5,6-di­hydro­benzimidazo[1,2-c]quinazoline moiety has been found to be disordered over two sets of sites, with occupancy ratio of 0.863 (2):0.137 (2), related by a pseudo-mirror plane approximately parallel to the (1 0 1) plane. Within the disordered components, the dihedral angle between the benzimidazole ring and the benzene ring of the quinazoline group is 14.28 (5) and 4.7 (3)°, respectively. The molecular conformation is enforced by an intra­molecular O—H···O hydrogen bond (Table 1). In the crystal, molecules form chains parallel to the [1 0 -1] direction *via* O—H···N hydrogen bonds.

### S2. Refinement

During the refinement of the disordered 5,6-di­hydro­benzimidazo[1,2-c]quinazoline moiety DELU and SIMU restraints were applied. The phenolic hydrogen atom was located in a difference Fourier map and refined freely. All other H atoms were calculated geometrically and refined using a riding model, with C—H = 0.93-0.98 Å, N—H = 0.86 Å, and with *U*_iso_(H) = 1.2 *U*_eq_(C, N) or 1.5 *U*_eq_(C) for methyl H atoms. A rotating model was used for the methyl group. Four outliers (-3 0 1, -7 0 1, 0 0 2, 0 0 6) were omitted in the last cycles of refinement.

### S3. Synthesis and crystallization

3-Hy­droxy-4-meth­oxy-benzaldehyde (5 mmol, 0.761 g) and 2-(2-amino­phenyl)-1H-benzimidazole (5 mmol, 1.046 g) were dissolved in ethanol in separate beakers, then the amine solution was added drop wise with stirring to the aldehyde solution in a round bottomed flask. The mixture was refluxed over 4h. The product was filtered and washed with hot ethanol and *n*-hexane, then dried out over 24 h under reduced pressure in a desiccator. Single crystals suitable for X-ray analysis were formed on slow evaporation of the ethanol solution. M. p.: 560-561 K; Yield: 97%. Anal. Cal. for C_21_H_17_N_3_O_2_ (fw: 343.43 g/mol); C, 73.56; H, 4.95; N, 12.23; Found: C, 73.90; H, 4.56; N, 12.24%. IR (KBr pellets ν_max_/cm^-1^): 3382 ν(OH), 1616 ν(C=N), 1497 ν(CH3, stre.), 1154 ν(C—O), 1127 ν(C—N).

### Crystal data

**Table:**

C_21_H_17_N_3_O_2_	*D*_x_ = 1.334 Mg m^−^^3^
*M**_r_* = 343.38	Melting point < 560 K
Monoclinic, *P*2_1_/*n*	Mo *K*α radiation, λ = 0.71073 Å
*a* = 9.7359 (7) Å	Cell parameters from 4581 reflections
*b* = 10.0822 (7) Å	θ = 3.1–27.8°
*c* = 17.4624 (13) Å	µ = 0.09 mm^−^^1^
β = 94.2958 (15)°	*T* = 294 K
*V* = 1709.3 (2) Å^3^	Block, colourless
*Z* = 4	0.29 × 0.20 × 0.12 mm
*F*(000) = 720	

### Data collection

**Table:**

Bruker APEXII CCD diffractometer	*R*_int_ = 0.030
φ and ω scans	θ_max_ = 30.1°, θ_min_ = 2.3°
19177 measured reflections	*h* = −13→13
4987 independent reflections	*k* = −14→14
3575 reflections with *I* > 2σ(*I*)	*l* = −23→24

### Refinement

**Table:**

Refinement on *F*^2^	752 restraints
Least-squares matrix: full	Hydrogen site location: mixed
*R*[*F*^2^ > 2σ(*F*^2^)] = 0.049	H atoms treated by a mixture of independent and constrained refinement
*wR*(*F*^2^) = 0.139	*w* = 1/[σ^2^(*F*_o_^2^) + (0.0534*P*)^2^ + 0.4546*P*] where *P* = (*F*_o_^2^ + 2*F*_c_^2^)/3
*S* = 1.05	(Δ/σ)_max_ < 0.001
4987 reflections	Δρ_max_ = 0.34 e Å^−^^3^
370 parameters	Δρ_min_ = −0.23 e Å^−^^3^

### Special details

**Table:**

Geometry. All e.s.d.'s (except the e.s.d. in the dihedral angle between two l.s. planes) are estimated using the full covariance matrix. The cell e.s.d.'s are taken into account individually in the estimation of e.s.d.'s in distances, angles and torsion angles; correlations between e.s.d.'s in cell parameters are only used when they are defined by crystal symmetry. An approximate (isotropic) treatment of cell e.s.d.'s is used for estimating e.s.d.'s involving l.s. planes.

### Fractional atomic coordinates and isotropic or equivalent isotropic displacement parameters (Å^2^)

**Table:**

	*x*	*y*	*z*	*U*_iso_*/*U*_eq_	Occ. (<1)
O1	0.93764 (14)	0.63506 (12)	−0.04088 (7)	0.0588 (4)	
O2	0.86896 (12)	0.89115 (10)	−0.05203 (6)	0.0491 (3)	
N1	0.59770 (15)	0.75237 (13)	0.35710 (8)	0.0396 (3)	0.863 (2)
N2	0.69523 (14)	0.63791 (13)	0.26610 (7)	0.0359 (3)	0.863 (2)
N3	0.57057 (16)	0.51245 (15)	0.17367 (9)	0.0468 (4)	0.863 (2)
H3B	0.5651	0.4327	0.1563	0.056*	0.863 (2)
C1	0.7995 (2)	0.6669 (2)	0.32075 (11)	0.0351 (4)	0.863 (2)
C2	0.9392 (3)	0.6376 (2)	0.32537 (13)	0.0478 (5)	0.863 (2)
H2A	0.9797	0.5919	0.2866	0.057*	0.863 (2)
C3	1.0146 (3)	0.6803 (3)	0.39098 (19)	0.0598 (7)	0.863 (2)
H3A	1.1089	0.6642	0.3960	0.072*	0.863 (2)
C4	0.9543 (3)	0.7462 (3)	0.44958 (16)	0.0597 (7)	0.863 (2)
H4A	1.0086	0.7713	0.4933	0.072*	0.863 (2)
C5	0.8155 (3)	0.7756 (2)	0.44454 (12)	0.0487 (5)	0.863 (2)
H5A	0.7753	0.8198	0.4839	0.058*	0.863 (2)
C6	0.7377 (2)	0.7363 (2)	0.37795 (13)	0.0375 (4)	0.863 (2)
C7	0.5774 (3)	0.6934 (7)	0.2911 (3)	0.0361 (7)	0.863 (2)
C8	0.4506 (2)	0.6765 (2)	0.24247 (13)	0.0403 (4)	0.863 (2)
C9	0.3310 (3)	0.7459 (2)	0.25541 (13)	0.0526 (5)	0.863 (2)
H9A	0.3318	0.8086	0.2945	0.063*	0.863 (2)
C10	0.2110 (3)	0.7218 (3)	0.2101 (2)	0.0635 (8)	0.863 (2)
H10A	0.1311	0.7688	0.2180	0.076*	0.863 (2)
C11	0.2111 (3)	0.6263 (4)	0.1525 (2)	0.0674 (9)	0.863 (2)
H11A	0.1300	0.6088	0.1226	0.081*	0.863 (2)
C12	0.3284 (3)	0.5578 (3)	0.13899 (14)	0.0559 (6)	0.863 (2)
H12A	0.3264	0.4942	0.1003	0.067*	0.863 (2)
C13	0.4515 (2)	0.5835 (2)	0.18347 (13)	0.0424 (4)	0.863 (2)
C14	0.7041 (5)	0.5718 (5)	0.1925 (2)	0.0344 (6)	0.863 (2)
H14B	0.7722	0.5004	0.1994	0.041*	0.863 (2)
N1X	0.5001 (9)	0.7439 (9)	0.3169 (5)	0.0386 (19)	0.137 (2)
N2X	0.5950 (10)	0.6217 (9)	0.2297 (5)	0.0419 (19)	0.137 (2)
N3X	0.8202 (12)	0.5396 (10)	0.2406 (5)	0.056 (2)	0.137 (2)
H3XB	0.8617	0.4652	0.2356	0.067*	0.137 (2)
C1X	0.4602 (9)	0.6321 (10)	0.2060 (7)	0.040 (2)	0.137 (2)
C2X	0.3809 (14)	0.5828 (12)	0.1429 (6)	0.053 (3)	0.137 (2)
H2XA	0.4212	0.5298	0.1071	0.063*	0.137 (2)
C3X	0.2413 (14)	0.6126 (16)	0.1331 (6)	0.059 (4)	0.137 (2)
H3XA	0.1882	0.5796	0.0908	0.071*	0.137 (2)
C4X	0.1810 (9)	0.6918 (15)	0.1865 (8)	0.052 (4)	0.137 (2)
H4XA	0.0876	0.7117	0.1799	0.063*	0.137 (2)
C5X	0.2603 (13)	0.7411 (10)	0.2496 (6)	0.054 (3)	0.137 (2)
H5XA	0.2200	0.7941	0.2853	0.065*	0.137 (2)
C6X	0.3999 (12)	0.7113 (10)	0.2594 (5)	0.036 (2)	0.137 (2)
C7X	0.611 (2)	0.683 (4)	0.2897 (19)	0.032 (3)	0.137 (2)
C8X	0.7465 (9)	0.6924 (9)	0.3393 (6)	0.036 (2)	0.137 (2)
C9X	0.7716 (11)	0.7555 (10)	0.4097 (7)	0.043 (3)	0.137 (2)
H9XA	0.7015	0.8027	0.4308	0.051*	0.137 (2)
C10X	0.9013 (14)	0.7482 (15)	0.4486 (6)	0.061 (5)	0.137 (2)
H10B	0.9180	0.7904	0.4957	0.073*	0.137 (2)
C11X	1.0059 (10)	0.6776 (18)	0.4170 (8)	0.071 (5)	0.137 (2)
H11B	1.0927	0.6727	0.4430	0.085*	0.137 (2)
C12X	0.9809 (11)	0.6144 (14)	0.3466 (7)	0.061 (4)	0.137 (2)
H12B	1.0509	0.5673	0.3255	0.074*	0.137 (2)
C13X	0.8512 (13)	0.6218 (9)	0.3077 (5)	0.045 (2)	0.137 (2)
C14X	0.712 (4)	0.588 (3)	0.1788 (18)	0.052 (6)	0.137 (2)
H14A	0.6815	0.5086	0.1499	0.062*	0.137 (2)
C15	0.75062 (14)	0.66702 (13)	0.13052 (7)	0.0347 (3)	
C16	0.82721 (14)	0.61424 (13)	0.07362 (8)	0.0360 (3)	
H16A	0.8526	0.5253	0.0760	0.043*	
C17	0.86614 (14)	0.69147 (13)	0.01369 (7)	0.0351 (3)	
C18	0.82827 (14)	0.82542 (13)	0.01053 (7)	0.0354 (3)	
C19	0.75414 (18)	0.87820 (14)	0.06726 (9)	0.0465 (4)	
H19A	0.7295	0.9673	0.0654	0.056*	
C20	0.71589 (18)	0.79913 (14)	0.12733 (9)	0.0475 (4)	
H20A	0.6664	0.8359	0.1656	0.057*	
C21	0.8361 (2)	1.02811 (16)	−0.05978 (11)	0.0623 (5)	
H21A	0.8729	1.0624	−0.1053	0.093*	
H21B	0.8754	1.0754	−0.0157	0.093*	
H21C	0.7379	1.0390	−0.0636	0.093*	
H1O1	0.969 (3)	0.692 (3)	−0.0666 (15)	0.096 (8)*	

### Atomic displacement parameters (Å^2^)

**Table:**

	*U*^11^	*U*^22^	*U*^33^	*U*^12^	*U*^13^	*U*^23^
O1	0.0862 (9)	0.0403 (6)	0.0563 (7)	0.0106 (6)	0.0472 (7)	0.0054 (5)
O2	0.0672 (7)	0.0399 (5)	0.0426 (6)	0.0027 (5)	0.0206 (5)	0.0083 (4)
N1	0.0473 (8)	0.0401 (7)	0.0333 (7)	0.0007 (6)	0.0145 (6)	−0.0021 (5)
N2	0.0400 (7)	0.0406 (7)	0.0284 (6)	0.0019 (6)	0.0117 (5)	−0.0006 (5)
N3	0.0548 (9)	0.0404 (7)	0.0466 (8)	−0.0107 (6)	0.0136 (6)	−0.0101 (6)
C1	0.0402 (10)	0.0353 (9)	0.0305 (8)	0.0008 (8)	0.0081 (7)	0.0053 (7)
C2	0.0433 (13)	0.0536 (11)	0.0471 (12)	0.0052 (10)	0.0077 (9)	0.0060 (9)
C3	0.0489 (12)	0.0677 (16)	0.0609 (17)	0.0029 (11)	−0.0089 (10)	0.0096 (13)
C4	0.0650 (18)	0.0624 (15)	0.0490 (13)	−0.0030 (14)	−0.0144 (11)	0.0055 (10)
C5	0.0674 (15)	0.0454 (10)	0.0328 (10)	−0.0047 (10)	0.0008 (9)	0.0020 (8)
C6	0.0496 (11)	0.0341 (9)	0.0297 (10)	−0.0012 (8)	0.0089 (8)	0.0027 (8)
C7	0.0403 (16)	0.0370 (13)	0.0331 (10)	0.0027 (15)	0.0174 (13)	0.0005 (8)
C8	0.0396 (10)	0.0447 (11)	0.0379 (11)	−0.0012 (8)	0.0108 (8)	0.0023 (8)
C9	0.0419 (12)	0.0642 (13)	0.0529 (12)	0.0060 (12)	0.0115 (11)	0.0099 (9)
C10	0.0392 (12)	0.085 (2)	0.067 (2)	0.0031 (13)	0.0073 (12)	0.0217 (16)
C11	0.0461 (12)	0.092 (2)	0.0631 (19)	−0.0165 (14)	−0.0052 (13)	0.0200 (16)
C12	0.0547 (15)	0.0652 (14)	0.0469 (11)	−0.0225 (12)	−0.0023 (10)	0.0043 (10)
C13	0.0451 (10)	0.0463 (11)	0.0363 (10)	−0.0126 (9)	0.0074 (8)	0.0041 (8)
C14	0.0465 (11)	0.0317 (9)	0.0272 (14)	0.0000 (8)	0.0162 (9)	0.0024 (9)
N1X	0.042 (4)	0.045 (4)	0.030 (4)	−0.001 (4)	0.009 (3)	−0.003 (3)
N2X	0.058 (4)	0.043 (4)	0.026 (4)	0.000 (4)	0.015 (3)	−0.003 (3)
N3X	0.075 (6)	0.053 (5)	0.042 (5)	0.030 (5)	0.026 (4)	0.017 (4)
C1X	0.052 (5)	0.037 (6)	0.032 (6)	−0.009 (5)	0.011 (4)	−0.015 (4)
C2X	0.063 (7)	0.063 (8)	0.035 (6)	−0.031 (7)	0.020 (5)	−0.013 (5)
C3X	0.074 (9)	0.068 (9)	0.037 (7)	−0.015 (8)	0.005 (7)	0.011 (5)
C4X	0.046 (7)	0.070 (10)	0.040 (8)	−0.009 (6)	−0.005 (5)	0.008 (6)
C5X	0.034 (6)	0.075 (8)	0.054 (7)	−0.001 (7)	−0.003 (6)	0.002 (6)
C6X	0.035 (5)	0.039 (6)	0.033 (5)	0.001 (5)	0.002 (5)	−0.005 (4)
C7X	0.036 (6)	0.030 (7)	0.030 (5)	−0.001 (7)	0.015 (6)	0.001 (4)
C8X	0.047 (5)	0.031 (5)	0.033 (5)	0.006 (4)	0.015 (4)	−0.005 (4)
C9X	0.050 (6)	0.043 (6)	0.035 (7)	−0.004 (5)	0.000 (5)	0.002 (5)
C10X	0.059 (10)	0.063 (8)	0.058 (7)	0.005 (8)	−0.021 (7)	0.014 (6)
C11X	0.063 (8)	0.079 (11)	0.067 (10)	0.003 (7)	−0.019 (7)	0.022 (8)
C12X	0.045 (7)	0.083 (9)	0.057 (8)	0.013 (6)	0.008 (5)	0.033 (6)
C13X	0.034 (5)	0.055 (7)	0.048 (5)	0.011 (5)	0.012 (4)	0.026 (4)
C14X	0.063 (8)	0.055 (13)	0.038 (10)	0.015 (9)	0.013 (6)	−0.021 (9)
C15	0.0395 (7)	0.0351 (6)	0.0306 (6)	0.0002 (5)	0.0092 (5)	−0.0015 (5)
C16	0.0412 (7)	0.0308 (6)	0.0374 (7)	0.0015 (5)	0.0127 (5)	0.0000 (5)
C17	0.0376 (7)	0.0356 (6)	0.0335 (6)	−0.0005 (5)	0.0121 (5)	−0.0036 (5)
C18	0.0420 (7)	0.0337 (6)	0.0311 (6)	−0.0016 (5)	0.0073 (5)	0.0012 (5)
C19	0.0646 (10)	0.0306 (6)	0.0468 (8)	0.0050 (6)	0.0198 (7)	−0.0008 (6)
C20	0.0670 (10)	0.0369 (7)	0.0415 (8)	0.0053 (7)	0.0244 (7)	−0.0053 (6)
C21	0.0813 (13)	0.0413 (8)	0.0660 (11)	−0.0010 (8)	0.0158 (9)	0.0155 (8)

### Geometric parameters (Å, º)

**Table:**

O1—C17	1.3473 (16)	N2X—C14X	1.53 (3)
O1—H1O1	0.80 (3)	N3X—C13X	1.449 (14)
O2—C18	1.3617 (16)	N3X—C14X	1.53 (3)
O2—C21	1.4215 (19)	N3X—H3XB	0.8600
N1—C7	1.299 (5)	C1X—C2X	1.3900
N1—C6	1.394 (2)	C1X—C6X	1.3900
N2—C1	1.372 (2)	C2X—C3X	1.3900
N2—C7	1.377 (3)	C2X—H2XA	0.9300
N2—C14	1.455 (5)	C3X—C4X	1.3900
N3—C13	1.384 (3)	C3X—H3XA	0.9300
N3—C14	1.446 (5)	C4X—C5X	1.3900
N3—H3B	0.8600	C4X—H4XA	0.9300
C1—C2	1.389 (3)	C5X—C6X	1.3900
C1—C6	1.392 (3)	C5X—H5XA	0.9300
C2—C3	1.382 (3)	C7X—C8X	1.53 (3)
C2—H2A	0.9300	C8X—C9X	1.3900
C3—C4	1.387 (4)	C8X—C13X	1.3900
C3—H3A	0.9300	C9X—C10X	1.3900
C4—C5	1.380 (4)	C9X—H9XA	0.9300
C4—H4A	0.9300	C10X—C11X	1.3900
C5—C6	1.397 (3)	C10X—H10B	0.9300
C5—H5A	0.9300	C11X—C12X	1.3900
C7—C8	1.455 (4)	C11X—H11B	0.9300
C8—C9	1.391 (3)	C12X—C13X	1.3900
C8—C13	1.394 (3)	C12X—H12B	0.9300
C9—C10	1.383 (4)	C14X—C15	1.24 (3)
C9—H9A	0.9300	C14X—H14A	0.9800
C10—C11	1.392 (4)	C15—C20	1.3744 (19)
C10—H10A	0.9300	C15—C16	1.3919 (18)
C11—C12	1.370 (4)	C16—C17	1.3801 (18)
C11—H11A	0.9300	C16—H16A	0.9300
C12—C13	1.403 (3)	C17—C18	1.4000 (19)
C12—H12A	0.9300	C18—C19	1.376 (2)
C14—C15	1.540 (4)	C19—C20	1.390 (2)
C14—H14B	0.9800	C19—H19A	0.9300
N1X—C7X	1.36 (2)	C20—H20A	0.9300
N1X—C6X	1.386 (12)	C21—H21A	0.9600
N2X—C7X	1.22 (3)	C21—H21B	0.9600
N2X—C1X	1.350 (13)	C21—H21C	0.9600
			
C17—O1—H1O1	109.3 (19)	C1X—C2X—H2XA	120.0
C18—O2—C21	118.18 (12)	C2X—C3X—C4X	120.0
C7—N1—C6	105.05 (18)	C2X—C3X—H3XA	120.0
C1—N2—C7	106.5 (2)	C4X—C3X—H3XA	120.0
C1—N2—C14	128.5 (2)	C5X—C4X—C3X	120.0
C7—N2—C14	124.9 (3)	C5X—C4X—H4XA	120.0
C13—N3—C14	120.3 (2)	C3X—C4X—H4XA	120.0
C13—N3—H3B	119.9	C4X—C5X—C6X	120.0
C14—N3—H3B	119.9	C4X—C5X—H5XA	120.0
N2—C1—C2	131.7 (2)	C6X—C5X—H5XA	120.0
N2—C1—C6	105.6 (2)	N1X—C6X—C5X	131.8 (10)
C2—C1—C6	122.63 (17)	N1X—C6X—C1X	108.2 (10)
C3—C2—C1	116.0 (2)	C5X—C6X—C1X	120.0
C3—C2—H2A	122.0	N2X—C7X—N1X	118 (2)
C1—C2—H2A	122.0	N2X—C7X—C8X	124.7 (18)
C2—C3—C4	122.2 (3)	N1X—C7X—C8X	117 (2)
C2—C3—H3A	118.9	C9X—C8X—C13X	120.0
C4—C3—H3A	118.9	C9X—C8X—C7X	128.1 (13)
C5—C4—C3	121.5 (2)	C13X—C8X—C7X	111.9 (13)
C5—C4—H4A	119.2	C8X—C9X—C10X	120.0
C3—C4—H4A	119.2	C8X—C9X—H9XA	120.0
C4—C5—C6	117.4 (2)	C10X—C9X—H9XA	120.0
C4—C5—H5A	121.3	C11X—C10X—C9X	120.0
C6—C5—H5A	121.3	C11X—C10X—H10B	120.0
C1—C6—N1	109.7 (2)	C9X—C10X—H10B	120.0
C1—C6—C5	120.2 (2)	C10X—C11X—C12X	120.0
N1—C6—C5	130.0 (2)	C10X—C11X—H11B	120.0
N1—C7—N2	113.1 (3)	C12X—C11X—H11B	120.0
N1—C7—C8	129.6 (2)	C13X—C12X—C11X	120.0
N2—C7—C8	117.3 (3)	C13X—C12X—H12B	120.0
C9—C8—C13	120.7 (2)	C11X—C12X—H12B	120.0
C9—C8—C7	122.0 (3)	C12X—C13X—C8X	120.0
C13—C8—C7	117.2 (3)	C12X—C13X—N3X	119.4 (10)
C10—C9—C8	120.0 (2)	C8X—C13X—N3X	119.9 (10)
C10—C9—H9A	120.0	C15—C14X—N3X	117 (3)
C8—C9—H9A	120.0	C15—C14X—N2X	122 (2)
C9—C10—C11	119.3 (2)	N3X—C14X—N2X	99.5 (18)
C9—C10—H10A	120.4	C15—C14X—H14A	105.7
C11—C10—H10A	120.4	N3X—C14X—H14A	105.7
C12—C11—C10	121.2 (2)	N2X—C14X—H14A	105.7
C12—C11—H11A	119.4	C14X—C15—C20	124.4 (14)
C10—C11—H11A	119.4	C14X—C15—C16	116.5 (15)
C11—C12—C13	120.0 (3)	C20—C15—C16	119.00 (12)
C11—C12—H12A	120.0	C20—C15—C14	123.2 (2)
C13—C12—H12A	120.0	C16—C15—C14	117.7 (2)
N3—C13—C8	119.5 (2)	C17—C16—C15	121.20 (12)
N3—C13—C12	121.6 (3)	C17—C16—H16A	119.4
C8—C13—C12	118.7 (2)	C15—C16—H16A	119.4
N3—C14—N2	106.2 (3)	O1—C17—C16	118.95 (12)
N3—C14—C15	113.9 (3)	O1—C17—C18	121.86 (12)
N2—C14—C15	112.1 (3)	C16—C17—C18	119.18 (12)
N3—C14—H14B	108.2	O2—C18—C19	126.10 (12)
N2—C14—H14B	108.2	O2—C18—C17	114.18 (11)
C15—C14—H14B	108.2	C19—C18—C17	119.71 (12)
C7X—N1X—C6X	100.1 (14)	C18—C19—C20	120.41 (13)
C7X—N2X—C1X	106.5 (13)	C18—C19—H19A	119.8
C7X—N2X—C14X	123.9 (19)	C20—C19—H19A	119.8
C1X—N2X—C14X	125.9 (15)	C15—C20—C19	120.48 (13)
C13X—N3X—C14X	118.6 (14)	C15—C20—H20A	119.8
C13X—N3X—H3XB	120.7	C19—C20—H20A	119.8
C14X—N3X—H3XB	120.7	O2—C21—H21A	109.5
N2X—C1X—C2X	133.2 (10)	O2—C21—H21B	109.5
N2X—C1X—C6X	106.8 (10)	H21A—C21—H21B	109.5
C2X—C1X—C6X	120.0	O2—C21—H21C	109.5
C3X—C2X—C1X	120.0	H21A—C21—H21C	109.5
C3X—C2X—H2XA	120.0	H21B—C21—H21C	109.5
			
C7—N2—C1—C2	−179.3 (4)	N2X—C1X—C6X—C5X	179.3 (9)
C14—N2—C1—C2	−4.0 (4)	C2X—C1X—C6X—C5X	0.0
C7—N2—C1—C6	1.7 (4)	C1X—N2X—C7X—N1X	0 (4)
C14—N2—C1—C6	176.9 (2)	C14X—N2X—C7X—N1X	159 (2)
N2—C1—C2—C3	−178.3 (2)	C1X—N2X—C7X—C8X	177 (3)
C6—C1—C2—C3	0.6 (3)	C14X—N2X—C7X—C8X	−24 (5)
C1—C2—C3—C4	1.2 (4)	C6X—N1X—C7X—N2X	−1 (4)
C2—C3—C4—C5	−1.6 (4)	C6X—N1X—C7X—C8X	−178 (2)
C3—C4—C5—C6	0.0 (4)	N2X—C7X—C8X—C9X	−177 (2)
N2—C1—C6—N1	−1.64 (19)	N1X—C7X—C8X—C9X	0 (4)
C2—C1—C6—N1	179.22 (17)	N2X—C7X—C8X—C13X	0 (4)
N2—C1—C6—C5	176.98 (18)	N1X—C7X—C8X—C13X	177 (2)
C2—C1—C6—C5	−2.2 (3)	C13X—C8X—C9X—C10X	0.0
C7—N1—C6—C1	0.9 (4)	C7X—C8X—C9X—C10X	177 (2)
C7—N1—C6—C5	−177.5 (4)	C8X—C9X—C10X—C11X	0.0
C4—C5—C6—C1	1.8 (3)	C9X—C10X—C11X—C12X	0.0
C4—C5—C6—N1	−179.9 (2)	C10X—C11X—C12X—C13X	0.0
C6—N1—C7—N2	0.2 (6)	C11X—C12X—C13X—C8X	0.0
C6—N1—C7—C8	178.9 (6)	C11X—C12X—C13X—N3X	−170.7 (9)
C1—N2—C7—N1	−1.2 (6)	C9X—C8X—C13X—C12X	0.0
C14—N2—C7—N1	−176.7 (3)	C7X—C8X—C13X—C12X	−177.5 (19)
C1—N2—C7—C8	179.9 (4)	C9X—C8X—C13X—N3X	170.6 (9)
C14—N2—C7—C8	4.5 (7)	C7X—C8X—C13X—N3X	−6.9 (19)
N1—C7—C8—C9	12.1 (9)	C14X—N3X—C13X—C12X	−152.5 (15)
N2—C7—C8—C9	−169.3 (4)	C14X—N3X—C13X—C8X	36.8 (17)
N1—C7—C8—C13	−165.5 (5)	C13X—N3X—C14X—C15	83 (3)
N2—C7—C8—C13	13.1 (7)	C13X—N3X—C14X—N2X	−50 (2)
C13—C8—C9—C10	0.8 (3)	C7X—N2X—C14X—C15	−85 (4)
C7—C8—C9—C10	−176.7 (4)	C1X—N2X—C14X—C15	70 (3)
C8—C9—C10—C11	0.9 (4)	C7X—N2X—C14X—N3X	46 (3)
C9—C10—C11—C12	−1.2 (4)	C1X—N2X—C14X—N3X	−159.1 (12)
C10—C11—C12—C13	−0.2 (4)	N3X—C14X—C15—C20	−109 (2)
C14—N3—C13—C8	−31.8 (3)	N2X—C14X—C15—C20	14 (4)
C14—N3—C13—C12	152.8 (2)	N3X—C14X—C15—C16	75 (3)
C9—C8—C13—N3	−177.67 (18)	N2X—C14X—C15—C16	−162.1 (19)
C7—C8—C13—N3	0.0 (4)	N3X—C14X—C15—C14	−60 (53)
C9—C8—C13—C12	−2.2 (3)	N2X—C14X—C15—C14	62 (52)
C7—C8—C13—C12	175.5 (4)	N3—C14—C15—C14X	−46 (54)
C11—C12—C13—N3	177.2 (2)	N2—C14—C15—C14X	−166 (54)
C11—C12—C13—C8	1.9 (3)	N3—C14—C15—C20	86.7 (3)
C13—N3—C14—N2	44.5 (3)	N2—C14—C15—C20	−33.8 (4)
C13—N3—C14—C15	−79.3 (3)	N3—C14—C15—C16	−90.8 (3)
C1—N2—C14—N3	154.53 (19)	N2—C14—C15—C16	148.6 (2)
C7—N2—C14—N3	−31.0 (5)	C14X—C15—C16—C17	174.9 (19)
C1—N2—C14—C15	−80.6 (3)	C20—C15—C16—C17	−1.3 (2)
C7—N2—C14—C15	93.9 (5)	C14—C15—C16—C17	176.3 (2)
C7X—N2X—C1X—C2X	−179 (2)	C15—C16—C17—O1	−178.56 (13)
C14X—N2X—C1X—C2X	22 (2)	C15—C16—C17—C18	0.3 (2)
C7X—N2X—C1X—C6X	1 (2)	C21—O2—C18—C19	1.7 (2)
C14X—N2X—C1X—C6X	−157.3 (16)	C21—O2—C18—C17	−179.45 (14)
N2X—C1X—C2X—C3X	−179.1 (11)	O1—C17—C18—O2	0.5 (2)
C6X—C1X—C2X—C3X	0.0	C16—C17—C18—O2	−178.36 (12)
C1X—C2X—C3X—C4X	0.0	O1—C17—C18—C19	179.39 (15)
C2X—C3X—C4X—C5X	0.0	C16—C17—C18—C19	0.5 (2)
C3X—C4X—C5X—C6X	0.0	O2—C18—C19—C20	178.31 (15)
C7X—N1X—C6X—C5X	−179.8 (19)	C17—C18—C19—C20	−0.4 (2)
C7X—N1X—C6X—C1X	2 (2)	C14X—C15—C20—C19	−175 (2)
C4X—C5X—C6X—N1X	−178.1 (11)	C16—C15—C20—C19	1.4 (2)
C4X—C5X—C6X—C1X	0.0	C14—C15—C20—C19	−176.1 (2)
N2X—C1X—C6X—N1X	−2.2 (8)	C18—C19—C20—C15	−0.5 (3)
C2X—C1X—C6X—N1X	178.5 (9)		

### Hydrogen-bond geometry (Å, º)

**Table:**

*D*—H···*A*	*D*—H	H···*A*	*D*···*A*	*D*—H···*A*
O1—H1*O*1···O2	0.80 (3)	2.25 (3)	2.6706 (16)	112 (3)
O1—H1*O*1···N1^i^	0.80 (3)	1.98 (3)	2.703 (2)	150 (3)
O1—H1*O*1···N1*X*^i^	0.80 (3)	2.18 (3)	2.873 (9)	145 (3)

Symmetry code: (i) *x*+1/2, −*y*+3/2, *z*−1/2.
