# Supplementary figures and images for: Crystal structure of 5-(5,6-di­hydro­benzo[4,5]imidazo[1,2-c]quinazolin-6-yl)-2-meth­oxy­phenol
Source: Acta Crystallogr E Crystallogr Commun. 2015 Nov 21;71(Pt 12):o971–2. doi: 10.1107/S2056989015021180 (PMC4719928; doi:10.1107/S2056989015021180)

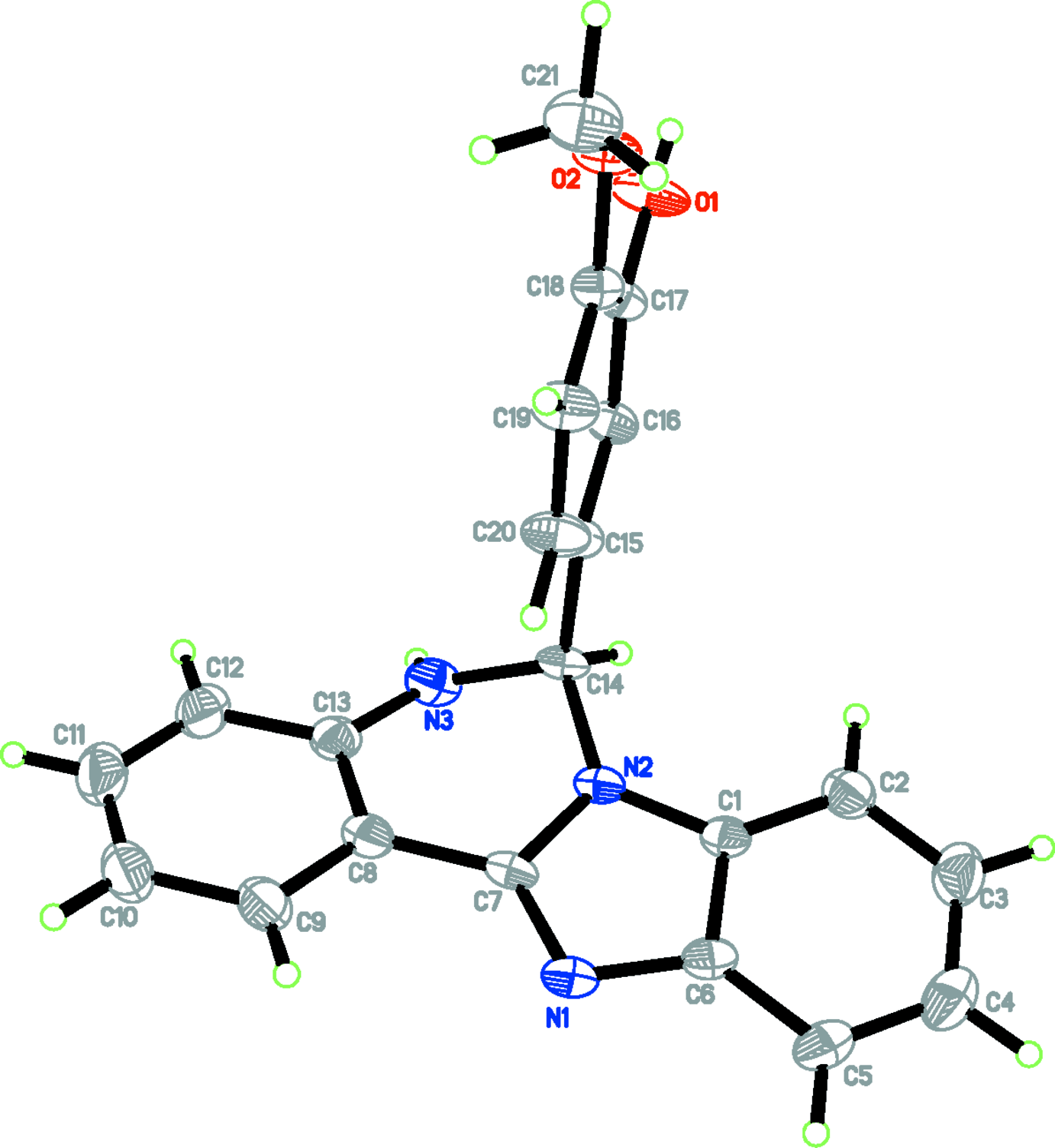

Supplement: Supplementary file 4 [file e-71-0o971-fig1.tif]

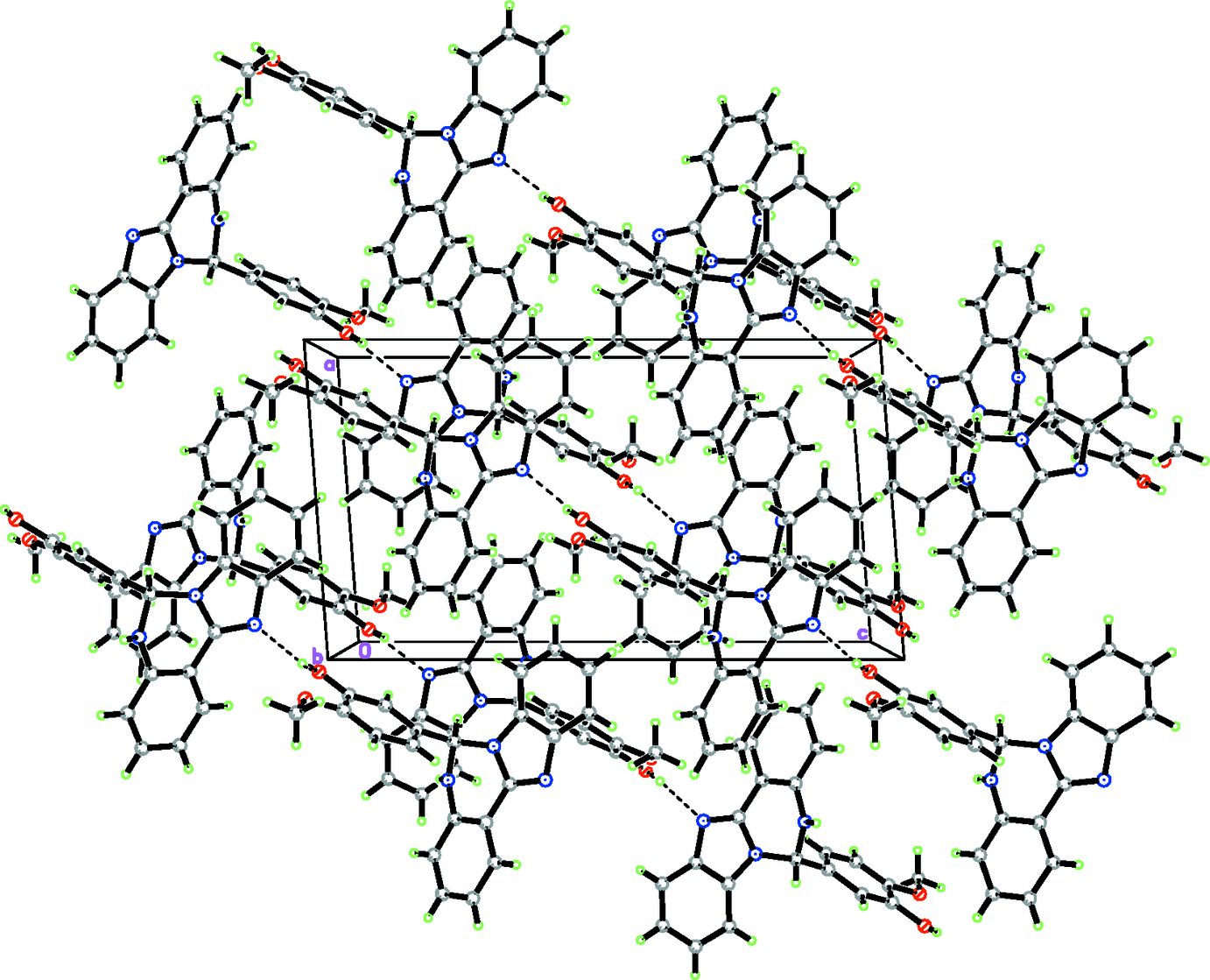

Supplement: Supplementary file 5 [file e-71-0o971-fig2.tif]
